# Microbiota characterization in *Blastocystis*-colonized and *Blastocystis*-free school-age children from Colombia

**DOI:** 10.1186/s13071-020-04392-9

**Published:** 2020-10-16

**Authors:** Sergio Castañeda, Marina Muñoz, Ximena Villamizar, Paula C. Hernández, Luis Reinel Vásquez, Raúl Yhossef Tito, Juan David Ramírez

**Affiliations:** 1 Grupo de Investigaciones Microbiológicas-UR (GIMUR), Departamento de Biología, Facultad de Ciencias Naturales, Universidad del Rosario, Bogotá, Colombia; 2 Maestría en Informática Biomédica, Facultad de Medicina, Universidad El Bosque, Bogotá, Colombia; 3 Laboratorio de Parasitología Molecular, Vicerrectoría de Investigaciones, Universidad El Bosque, Bogotá, Colombia; 4 Centro de Estudios en Microbiología y Parasitología (CEMPA), Departamento de Medicina Interna, Facultad de Ciencias de la Salud, Universidad del Cauca, Popayán, Colombia; 5 Department of Microbiology and Immunology, Rega Institute, KU Leuven, University of Leuven, Leuven, Belgium

**Keywords:** *Blastocystis*, Human microbiome, Gut microbiota alteration, Microbial diversity, Intestinal protozoans

## Abstract

**Background:**

*Blastocystis* is a protist that lives in the intestinal tract of a variety of hosts, including humans. It is still unclear how *Blastocystis* causes disease, which presents an ongoing challenge for researchers. Despite the controversial findings on the association between *Blastocystis* and clinical digestive manifestations, there is currently no consensus as to whether this protozoan actually behaves as a pathogen in humans. Furthermore, the relationship between *Blastocystis* and the intestinal microbiota composition is not yet clear. For that reason, the aim of this study was to identify if colonization by *Blastocystis* is related to changes in the diversity and relative abundance of bacterial communities, compared with those of *Blastocystis*-free individuals in a group of Colombian children.

**Methods:**

We took stool samples from 57 school-aged children attending a daycare institution in Popayán (Southwest Colombia). Whole DNA was extracted and examined by *16S*-rRNA amplicon-based sequencing. *Blastocystis* was detected by real time PCR and other intestinal parasites were detected by microscopy. We evaluated if *Blastocystis* was associated with host variables and the diversity and abundance of microbial communities.

**Results:**

The composition of the intestinal bacterial community was not significantly different between *Blastocystis*-free and *Blastocystis*-colonized children. Despite this, we observed a higher microbial richness in the intestines of children colonized by *Blastocystis*, which could, therefore, be considered a benefit to intestinal health. The phylum Firmicutes was the predominant taxonomic unit in both groups analyzed. In *Blastocystis*-free individuals, there was a higher proportion of Bacteroidetes; similarly, in children colonized by *Blastocystis*, there was a higher relative abundance of the phylum Proteobacteria; however, no statistically significant differences were found between the comparison groups.

**Conclusions:**

The presence of *Blastocystis* showed a decrease in *Bacteroides*, and an increase in the relative abundance of the genus *Faecalibacterium*. It was also evident that the presence of *Blastocystis* was unrelated to dysbiosis at the intestinal level; on the contrary, its presence did not show statistically differences in the intestinal microbiota composition. Nevertheless, we believe that *Blastocystis* plays a role in the ecology of the intestinal microbiota through its interaction with other microbial components.
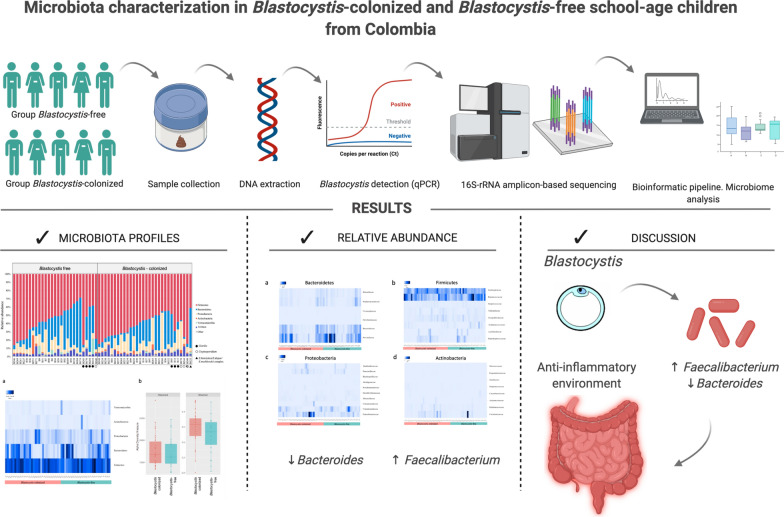

## Background

*Blastocystis* is a cosmopolitan enteric protozoan found in the digestive tract of humans, animals, birds, reptiles and amphibians. Its prevalence can exceed 5% in industrialized countries and may be between 30% and 100% in developing countries [1, 2]. Its pathogenic role is still controversial because it has been found in both symptomatic and asymptomatic individuals [3–5]. Some studies have suggested that this protozoan is associated with clinical symptoms, such as nausea, anorexia, flatulence, and acute or chronic diarrhea [6, 7]. An association of *Blastocystis* with irritable bowel syndrome and extra-intestinal manifestations, such as urticaria, has also been reported [8]. Because of its genetic diversity, which has mainly been established from molecular studies of the small subunit *18S* ribosomal RNA gene [9–11], *Blastocystis* has been classified into multiple subtypes (STs), including at least 17 currently known STs [7, 11–15]. A recent study revealed that the *Blastocystis* STs in the Colombian population are mainly ST1 (34%) and ST2 (23%) and to a lesser extent ST3 (11.4%), ST4 (0.8%), ST6 (19.8%), and ST8 (10.5%) [16].

Microbiome studies in patients colonized by *Blastocystis* are controversial, even though different studies have found that *Blastocystis* causes mainly digestive clinical manifestations, there is currently no agreement as to whether the protozoan actually behaves as a pathogen in humans. Studies carried out in developing countries have sought to establish possible changes in the intestinal microbiota based on the presence of this protozoan [17]. Research carried out on French patients with irritable bowel syndrome (IBS) discovered that *Blastocystis* was twice as prevalent in individuals with the disease than in the healthy population. Additionally, the incidence of *Blastocystis* was associated with a significant change in the composition of the gut microbiota, in which a decrease in “healthy” bacteria, mainly of the genus *Bifidobacterium*, was evidenced [5]. In contrast, a study carried out on the French population also revealed a possible relationship between the presence of *Blastocystis* and an increase in the diversity and abundance of intestinal microbiota, mainly observed as an increase in the class Clostridia and a decrease in the family *Enterobacteriaceae*, which suggested that *Blastocystis* is associated with a healthy gut microbiota [18].

During an investigation of Swedish adults comparing the features of *Blastocystis* infection with changes in the intestinal microbiota in travelers of this nationality, Forsell et al. [19] observed a probable relationship between the protozoan and the predominance of a healthy microbiota. Possible changes in the intestinal microbiota were evaluated in patients from Toowoomba, Australia and, despite the fact that some changes were observed that were mainly related to an increase in Firmicutes and a decrease in Bacteroidetes, no significant changes were observed in patients with respect to healthy individuals [20]. These contradictory findings have been attributed to the genetic diversity of the protozoan. A recent study that implemented *in vivo* and *in vitro* techniques found that *Blastocystis* ST7 potentially leads to an imbalance in the intestinal microbiota, suggesting that the ST shows pathogenicity *via* microbiotic modulation, thus providing a contrary position to the increasing reports on the commensal nature of this ubiquitous parasite [4, 21].

From the above evidence, it can be seen that the relationship between *Blastocystis* and the intestinal microbiota remains unclear and that it presents heterogeneous results. Moreover, no studies have been carried out in Colombia or even in South America to describe the composition of the intestinal microbiota in individuals colonized with this protozoan. Therefore, the objective of this study was to identify if *Blastocystis* colonization is related to changes in the diversity and relative abundance of bacterial communities in children. The intestinal compositions of *Blastocystis-*free and -colonized children from a rural area in southwest Colombia were compared.

## Methods

### Population study

The present study was carried out on samples obtained during the study by Villamizar et al. [16]. In this descriptive cross-sectional study, school-aged children (12 to 54 months-old) who attended a nursery school located in commune eight in Popayán, Cauca, Southwest Colombia were included. All samples were used for the identification of intestinal protozoans by conventional and molecular methods. *Blastocystis*, *Giardia duodenalis*, *Cryptosporidium* spp., and the *Entamoeba histolytica*/*dispar*/*moshkovskii* complex were identified by microscopy, quantitative PCR (qPCR), and conventional PCR.

From a survey of the parents of the participants selected for the present study, the following information was collated: intestinal discomfort; socioeconomic stratum (in Colombia, the strata are numbered 1 to 6 according to monthly income, strata 1–2 are considered low income, 3–4 middle income, and 5–6 high income); place of residence; age; sex; number of children in the house; monthly income; type of property; type of apartment; type of wall; availability of utilities; water quality; presence and number of pets; fecal disposal habits; hand washing habits; and garbage storage/disposal procedures.

Identification of the presence of *Blastocystis* was confirmed by both microscopy and qPCR, as reported elsewhere [16]. Based on the above results, two groups were selected from a non-probability sample: one of *Blastocystis*-colonized and the other of non-colonized children. We used 30 samples from the first group and 27 samples from the second. All samples (*n* = 57) were analyzed by amplicon-based sequencing of the *16S* rRNA gene to determine the intestinal bacteriome.

### *16S* rRNA-amplicon-based sequencing

All samples (*n* = 57) were subjected to bacterial diversity characterization by amplicon sequencing of the *16S* rRNA gene using the Illumina HiSeq platform. Primers 515-F (5ʹ-GTG CCA GCM GCC GCG GTA A-3ʹ) and 806-R (5ʹ-GGA CTA CHV GGG TWT CTA AT-3ʹ) [22] were used because the V4 hypervariable region has been reported to be the most informative region for describing bacterial communities. For the sequencing process, microbial amplicon libraries were built and sequenced until a minimum expected raw depth of 500 thousand reads per sample was reached.

### Microbiome analysis

Preparation was performed to extract barcodes and primers from the resulting paired-end sequences using the Quantitative Insights Into Microbial Ecology (QIIME) analysis program [23]. Using DADA2, the quality profile of the reads was evaluated to select the length with a quality greater than 30 and, thus, minimize erroneous reads. DADA2 was also used to determine the central sample inference algorithm of the reads to infer amplicon sequence variants (ASV). For this, duplications were eliminated by combining all identical sequencing reads into single sequences. Chimeras were removed before taxonomic allocation. Finally, taxonomic assignment was performed using the sequences of the Silva v132 formatted reference database [24]. The R *phyloseq* package was used to carry out analyzes of microbial diversity [25]. Using this package for the calculation of the diversity and abundance indices and the construction of the respective graphs, a phyloseq object was constructed that synthesized the different types of sequencing data. This was then used to calculate the diversity metrics in the *Blastocystis*-free and -colonized groups and the presence of co-infections with other parasites (*Giardia*, *Cryptosporidium* and the *Entamoeba histolytica*/*dispar*/*moshkovskii*). Based on the results, the most representative phyla and families in terms of relative abundance were defined for the *Blastocystis*-free and -colonized groups. Also, some genera were considered as biomarkers (previously reported as associated with intestinal health) for comparing the abundance and diversity of the microbiota between the groups.

### Statistical analysis

A descriptive analysis of the sociodemographic variables collated in the survey of the 57 evaluated individuals was performed. The quantitative variables (age) were summarized in terms of means and standard deviation, and the qualitative variables (gender, presence of diarrhea, pet ownership, source and quality of water, hand washing, and results of *Blastocystis* and *Giardia* identification) were summarized in frequencies and proportions. The number of reads in each sample was normalized using the average sequence depth. As indicators for the comparisons between the groups with and without *Blastocystis*, relative abundance and alpha diversity metrics, i.e. richness (defined as the total number of ASVs or species recorded) and Shannon index, were used [26]. The alpha diversity indices of children colonized by *Blastocystis* were compared with *Blastocystis*-free individuals using the non-parametric Mann-Whitney-Wilcoxon test. To explore the differences in the general composition of the microbial community between the two groups of children (beta diversity), the Bray-Curtis taxonomic distances were calculated. Likewise, using the *Phyloseq* package in R, a principal coordinates analysis diagram was produced, in which the Bray-Curtis dictations were used between the samples to visualize the behavior of the groups. Statistical analyzes were carried out using the R software [25] (RStudio Team 2019). For all continuous values, normality hypotheses were evaluated using the Shapiro-Wilk test. All tests of significance were two-tailed, and *P*-values < 0.05 were considered statistically significant.

## Results

### Demographic variables and *Blastocystis* STs

We evaluated samples from 57 participants, of which 46% were female and 54% male. The average age of the children was 47 months (± 11 months), and 35% (*n* = 20) of the participants reported having a pet, mainly dogs (75%) and cats (25%). In all, 98% (*n* = 56) had a treated water aqueduct. Additionally, the participants reported that they consumed boiled water (88%) or a mix of both boiled water and non-boiled water (13%). All the participants recorded the use of a toilet for the elimination of excreta and always washed their hands after use. The parasitological microscopy analysis showed the presence of *Blastocystis* in 22 participants (39%); however, molecular qPCR analysis identified a total of 30 individuals colonized by *Blastocystis* (53%). Similarly, microscopy was positive for *Giardia* in four participants (7%); therefore, confirmation by qPCR was carried out, and a total of 8 individuals (14%) were positive for the parasite.

### Relative abundance of microbiota in *Blastocystis*-free and -colonized individuals

**Fig. 1.**
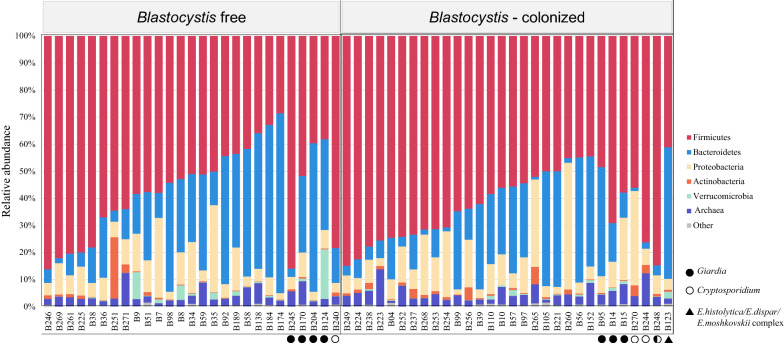
Bar chart of relative abundance at the phylum level between study participants. Likewise, the subjects that presented co-infection with other protozoans are shown

A total of 100,766,638 reads was obtained from the study population, with an average of 868,677.9 reads per sample, by sequencing the *16S* rRNA gene fragment (Additional file 1: Table S1). After pre-processing, consisting of high-quality filtering, the final number of reads was 95,814,972 with an average number of reads per sample of 825,991.1 (Additional file 1: Table S1). Subsequently, the composition of intestinal microbiota among participants with and without *Blastocystis* was evaluated at the phylum level (Fig. 1). It was clear that Firmicutes was the dominant phylum in all the groups, generally followed by Bacteroidetes and Proteobacteria and Actinobacteria.

**Fig. 2.**
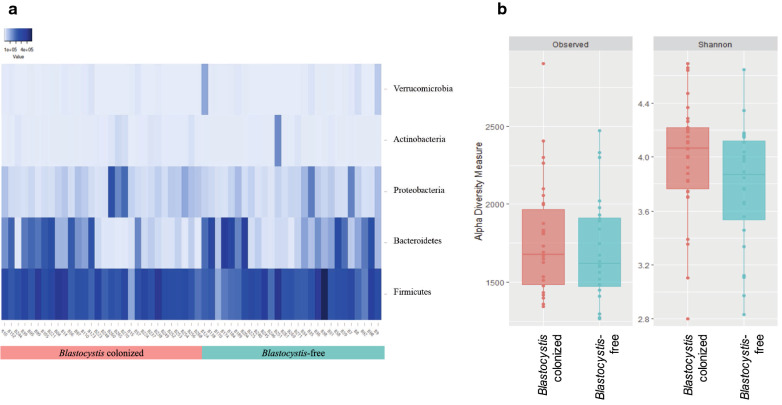
Heatmap of relative abundance at the phylum level between individuals colonized or not colonized by *Blastocystis* (**a**) and boxplot of observed OTU-richness and Shannon diversity indices distinguishing between participants colonized or not colonized by *Blastocystis* (**b**). Statistical analyzes were performed using the Mann-Whitney-Wilcoxon (MWW) test to compare *Blastocystis*-colonized and *Blastocystis*-free groups. Plotted are interquartile ranges (IQRs; boxes), medians (lines in the boxes), and the lowest and highest values within 1.5 times IQR from the first and third quartiles (whiskers above and below the boxes)

Analysis of relative abundance showed there was similarity between the phylum proportions of the evaluated groups (Fig. 2a). The phylum Firmicutes represented the highest proportion of intestinal bacteria in the different groups (t-test, *P* = 0.23). In the *Blastocystis*-free individuals, there was a higher proportion of Bacteroidetes compared with colonized individuals (Mann-Whitney-Wilcoxon test, *P* = 0.07). Similarly, bacteria of the phylum Proteobacteria showed a higher relative abundance in the *Blastocystis*-colonized group (Mann-Whitney-Wilcoxon test, *P* = 0.24); however, in both cases, no statistically significant differences were found.

**Fig. 3.**
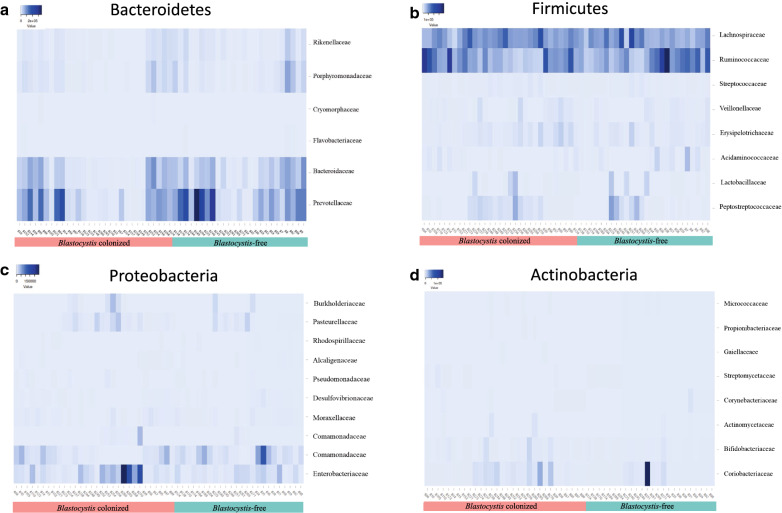
Heatmap of relative abundance of principal families between *Blastocystis*-colonized and free individuals related to phylum Bacteroidetes (**a**), phylum Firmicutes (**b**), phylum Proteobacteria (**c**) and phylum Actinobacteria (**d**)

Subsequently, the composition of the intestinal microbiota was identified at the level of the families with the greatest abundance belonging to the main phyla found (Fig. 3). Analysis and comparison of relative abundances was carried out for all the cases, and the groups were compared in terms of diversity based on the observed richness and the Shannon index. In the case of the phylum Bacteroidetes, the families with the highest relative abundance were identified, which corresponded to *Prevotellaceae*, *Bacteroidaceae*, *Porphyromonadaceae*, *Rikenellaceae*, *Flavobacteriaceae* and *Cryomorphaceae* (Fig. 3a). When comparing the study groups with respect to these families, no significant differences were found. In the case of the phylum Firmicutes, the families with the highest relative abundance were as follows (in descending order): *Ruminococcaceae*, *Lachnospiraceae*, *Peptostreptococcaceae*, *Erysipelotrichaceae*, *Lactobacillaceae*, *Acidaminococcaceae*, *Veillonellaceae* and *Streptococcaceae* (Fig. 3b). No differences were observed at this taxonomic level in terms of abundance and diversity. The following families were identified for the phylum Proteobacteria: *Enterobacteriaceae*, *Sutterellaceae*, *Pasteurellaceae*, *Burkholderiaceae*, *Moraxellaceae*, *Comamonadaceae*, *Desulfovibrionaceae*, *Pseudomonadaceae*, *Alcaligenaceae* and *Rhodospirillaceae* (Fig. 3c). No differences were identified between the groups evaluated in relation to these bacterial families. Regarding the phylum Actinobacteria, it was evident that the families with the highest relative abundance were Coriobacteriaceae, Bifidobacteriaceae, Actinomycetaceae, Corynebacteriaceae, Streptomycetaceae, Propionibacteriaceae, Micrococcaceae and Gaiellaceae (Fig. 3d). As in the previous cases, there were no significant differences between the evaluated groups and the bacterial composition referring to these families.

### Diversity indices in *Blastocystis*-free and -colonized individuals

To determine if there were changes in the bacterial diversity among the evaluated samples, we calculated the observed richness and Shannon indices. The composition of the intestinal bacterial community was not significantly different between *Blastocystis*-free and -colonized. When plotted graphically, there was an apparent increase in diversity in individuals colonized by *Blastocystis*; however, when the non-parametric Mann-Whitney-Wilcoxon test was used to compare *Blastocystis*-free *versus Blastocystis*-colonized participants, no differences in diversity were identified. The differences in intestinal microbiota composition among the two groups in terms of observed richness (Mann-Whitney-Wilcoxon test, *P* = 0.626) and Shannon index (Mann-Whitney-Wilcoxon test, *P* = 0.07) did not reach significant levels (Fig. 2b).

Because *Blastocystis* identification was performed together with *Giardia* identification, we were able to analyze how the presence of co-infections related to changes in diversity. Initially, the intestinal microbiota composition was determined in those children colonized by *Blastocystis* (*n* = 26), *Giardia* (*n* = 4), *Blastocystis-Giardia* co-infection (*n* = 4), and those without any protozoans (*n* = 23) (Fig. 1, Additional file 2: Figure S1). Despite that no statistically significant differences were found, graphically we observed in all cases, the highest proportion of the intestinal microbiome comprised the phylum Firmicutes (ANOVA test, *P* = 0.74). *Giardia* infection was related to a higher relative abundance of the phylum Bacteroidetes compared with the other groups (ANOVA test, *P* = 0.49). For individuals colonized by *Blastocystis*, as evidenced in Fig. 2a, the proportion of Bacteroidetes was lower than in those free of *Blastocystis* (Mann-Whitney-Wilcoxon test, *P* = 0.07). In individuals with *Blastocystis-Giardia* co-infections, the proportion of Bacteroidetes was lower than in the remaining groups (ANOVA test) and there was an increase in the proportion of Firmicutes (ANOVA test, *P* = 0.74) and Proteobacteria (Kruskal-Wallis H-test, *P* = 0.56) bacteria. However, Kruskal-Wallis diversity analysis of the groups found no significant differences in the observed wealth (Kruskal-Wallis H-test, *P* = 0.137) and Shannon indices (Kruskal-Wallis H-test, *P* = 0.290) (Additional file 2: Figure S1).

### Microbiota biomarkers in *Blastocystis*-free and -colonized individuals

**Fig. 4.**
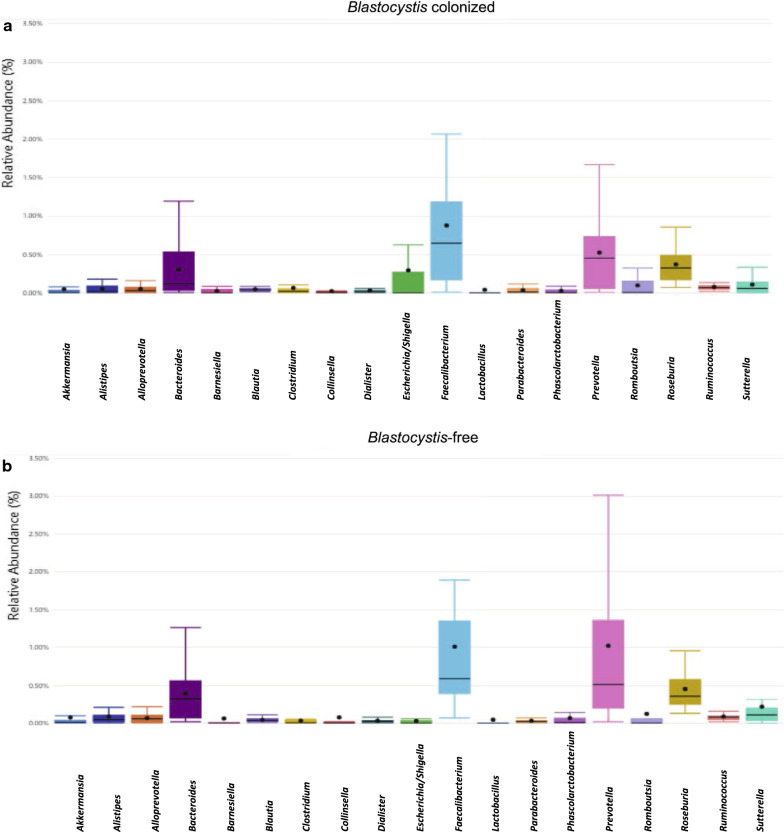
Boxplot of relative abundance of principal genera between *Blastocystis*-colonized (**a**) and non-colonized individuals (**b**). Plotted are interquartile ranges (IQRs; boxes), medians (lines in the boxes), and the lowest and highest values within 1.5 times IQR from the first and third quartiles (whiskers above and below the boxes)

The main bacterial genera of intestinal microbiomes were compared between individuals with and without *Blastocystis*. Initially, the relative abundance of the main bacterial groups of the intestinal microbiota was evaluated by genus (Fig. 4). The composition of the main bacterial groups of the intestinal microbiota was not significantly different between *Blastocystis*-free and -colonized in all cases. However, interestingly, we observed lower abundances of the genera *Bacteroidetes* and *Prevotella* in the *Blastocystis* group, while there was a higher abundance of *Faecalibacterium* bacteria in this group. Additionally, to identify changes in diversity, the observed richness was calculated for each of the following genera: *Bacteroides*, *Faecalibacterium*, *Prevotella*, *Roseburia*, *Ruminococcus* and *Akkermansia*. The descriptive results suggests there was greater richness of the genus *Bacteroides* and *Prevotella* (both genera belonging to the phylum Bacteriodetes) in the *Blastocystis*-free group, which corresponds to what was previously shown; nevertheless, the Mann-Whitney-Wilcoxon test did not find significant differences between the *Blastocystis-*colonized and non-colonized groups (Mann-Whitney-Wilcoxon test, *P* = 0.34 and *P* = 0.62, respectively). With regard to *Faecalibacterium*, *Roseburia*, *Ruminococcus* and *Akkermansia*, no characteristic changes were evidenced graphically and there were no statistically significant differences (Mann-Whitney-Wilcoxon test, *P* = 0.355, *P* = 0.681, *P* = 0.630, and *P* = 0.68, respectively).

**Fig. 5.**
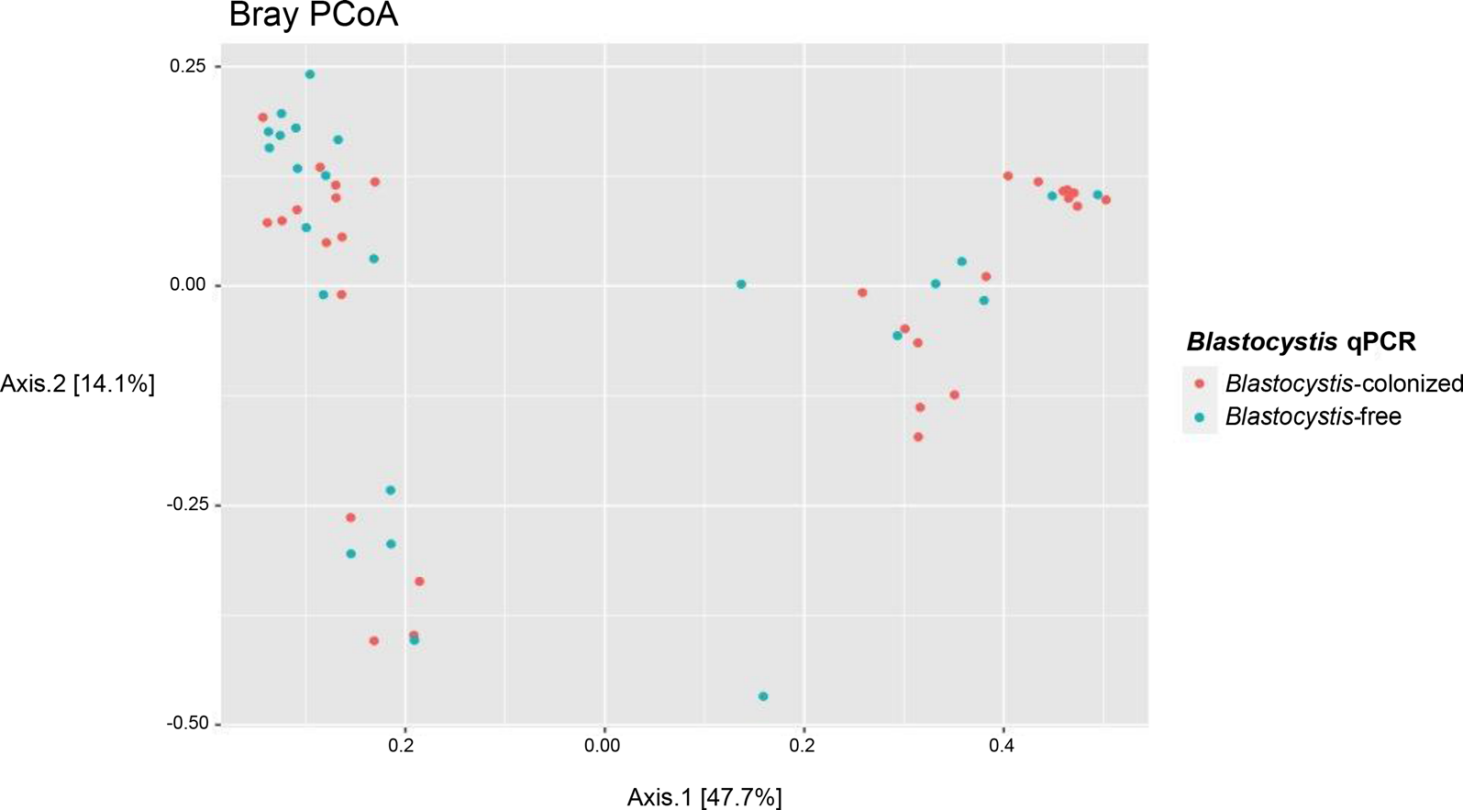
Principal coordinates analysis ordination of Bray-Curtis distance of the microbial communities in *Blastocystis*-colonized (red) and *Blastocystis*-free (blue) participant samples

To explore differences in the general composition of the microbial community between the two groups of participants (beta diversity), the Bray-Curtis dissimilarity distances were calculated, and a principal coordinates analysis diagram was produced (Fig. 5). The existence of 3 groups can be observed in the PCoA. Evaluating the different variables collected from the patients included in the present study, both biological and epidemiological, it was not possible to identify which variable explains this grouping. Regarding the presence or absence of *Blastocystis*, no point groupings were observed.

## Discussion

To the best of our knowledge, this is the first study of Colombian school-aged children to evaluate the diversity and abundance of intestinal bacterial communities in relation to the presence of *Blastocystis*. None of the evaluated individuals with *Blastocystis* (*n* = 30; 53%) had gastrointestinal symptoms associated with diarrhea, which is consistent with the undetermined pathogenic potential of this protozoan in humans [17]. Similarly, it is important to highlight that other intestinal protozoans were identified in the analyzed samples, including *Cryptosporidium*, *Entamoeba coli*, *Endolimax nana*, and the *Entamoeba histolytica*/*moshkovskii*/*dispar* complex [16]. However, because a subsample was taken, they were not considered in the analyzes carried out in this study. Additionally, the results highlight how molecular tests offer the diagnostic advantage of identifying a broad range of intestinal parasites [16]. In this study of *Blastocystis*, conventional methods identified 22 individuals with the protozoan, while qPCR identified 30 colonized individuals.

The evaluation of intestinal microbiota of individuals with and without *Blastocystis* has generated great interest in the scientific community because it may explain the pathogenic potential, innocuousness, or even beneficial characteristics this protozoan may have in humans [17]. In the present study, the composition of the intestinal bacterial community was not significantly different between *Blastocystis*-free and -colonized, nevertheless we observed, at least descriptively, differences among the groups of children with and without *Blastocystis*, from the perspective of the abundance and diversity of the intestinal microbiota, that suggest possible changes related to *Blastocystis* presence that must be verified with prospective studies. It was evident that the highest relative abundant phyla were Firmicutes, Bacteroidetes, Proteobacteria and Actinobacteria for all the groups evaluated (Fig. 1), which agrees with previously reported findings [18, 27–31]. Despite not identifying statistically significant differences, in the individuals colonized by *Blastocystis*, there was a probably lower abundance of Bacteroidetes and an increase in Proteobacteria (Fig. 2a). This is consistent with the analysis at the family level, where an apparent decrease in the relative abundance of *Bacteroideceae* and *Prevotellaceae*, belonging to the phylum Bacteroidetes, was observed in the group of subjects colonized by *Blastocystis* (Fig. 3a). Likewise, it is consistent with that observed in the phylum Proteobacteria, where the family *Enterobacteriaceae* shows a probable increase in abundance in *Blastocystis*-colonized subjects (Fig. 3c). This reinforces the theory proposed by Gabrielli et al. [32], in which ST3 is associated with a healthy microbiota and may be related to a greater richness of intestinal microbiota. Although there were no statistically significant changes between the groups with and without *Blastocystis*, the results suggest a greater richness and diversity of intestinal microbiota in the colonized individuals (Fig. 2b). These results are consistent with previous studies that have identified a greater richness in individuals with *Blastocystis*; however, these studies have been mainly performed in European populations [18, 19, 33]. It is important to highlight that, in the present descriptive study; non-probability sampling was carried out with a relatively small sample size. Therefore, prospective longitudinal and animal model studies are required that allow for a larger sample size to identify statistically significant associations and to corroborate these hypotheses.

Additional analyzes were performed to assess how the composition of the gut microbiota was related to the presence of co-infections. It was found that the presence of *Blastocystis*, both as the only protozoan and with a *Giardia* co-infection, was potentially associated with a lower relative abundance of *Bacteroidetes* (Additional file 2: Figure S1a). The diversity indices suggest there was an increase in the diversity of the intestinal microbiota of children with *Blastocystis*, as well as of those with *Blastocystis* + *Giardia* co-infection (Additional file 2: Figure S1b). These results agree with a study carried out on the Colombian population, which showed that *Giardia* infection was related to a decrease in the richness and diversity of intestinal microbiota, while *Blastocystis*, which was evaluated together with some helminths, was associated with increased richness [34]. Our results suggest that the presence of *Blastocystis* may be related to an increase in richness and that, in the case of *Giardia* co-infection, *Blastocystis* may have a beneficial effect on the level of intestinal microbiota richness, thus compensating for the impact of the infection. Nevertheless, because of the small sample size and this being a descriptive study, further prospective studies are required to establish this association.

The next level of analysis of the intestinal microbiota composition was the identification of the main genera present in the individuals with and without *Blastocystis*. The major genera were used as biomarkers to compare the groups in terms of diversity and abundance. Despite that, we did not find significant differences, interestingly, there was a lower relative abundance of *Bacteroides* and *Prevotella* and a higher relative abundance of *Faecalibacterium* bacteria in individuals with *Blastocystis* (Fig. 4). Additionally, a diversity analysis of the genera with the highest abundance was performed and the results of the groups compared. No statistically significant differences were found among the groups. However, the descriptive results do suggest there was a slight decrease in the richness of *Bacteroides* and *Prevotella* and an increase in the genus *Faecalibacterium* in individuals colonized by *Blastocystis*. These results are consistent with what has been previously reported by other research groups. A metagenomic study to establish the association between *Blastocystis* and microbial communities [33] found that the presence of this protozoan negatively correlated with the genus *Bacteroides* of the intestinal microbiome, thus suggesting a possible association between the bacterial component of the microbiota and the presence of *Blastocystis*. Additionally, this study found that this protozoan could be correlated with greater intestinal microbiota richness. A study of the Swedish population showed that the presence of *Blastocystis* (mainly ST4) was associated with a decrease in the relative abundance of the genus *Bacteroides* in individuals colonized by *Blastocystis*, indicating that this inverse relationship may be due to factors such as nutrient competition, competition for a specific ecological niche in the intestine, differences in the ability to use dietary nutrients, or even a direct negative effect of *Blastocystis* on *Bacteroides* [19]. Tito et al. [4] carried out a study using samples from the Flemish gut Flora Project, which included healthy individuals and individuals with IBD, and found greater diversity and richness of the intestinal microbiota consistent with the results of our study. Similarly, the authors identified a decrease in the relative abundance of *Bacteroides* and *Akkermansia*.

Interestingly, *Bacteroides* is one of the most abundant microorganisms in the human intestine and is involved in various physiological processes in the host [35, 36]. *Bacteroides* has been found to fulfill highly important functions, such as the metabolism of polysaccharides, which is fundamental to host adaptation to diet; the development of the immune system by inducing the growth of regulatory T cells; and the expression of cytokines that regulate inflammatory processes. Furthermore, they have been proposed to play a fundamental role in the ecology of the intestinal microbiota, maintaining interactions that allow modulation of other bacterial genera that fulfill relevant functions in the host. Similarly, a decrease in *Bacteroides* may be linked to inflammatory bowel disease, such as Crohn’s disease and ulcerative colitis [35–37]. Nonetheless, *Bacteroides* demonstrates the heterogeneity that microorganisms can have in terms of their relationship with, and impact on, their hosts because, despite being characterized as having a commensal and symbiotic nature, *Bacteroides* is known to also behave as a pathobiont [36]. It is important to note that bacteria of the genus *Bacteroides* maintain a complex and generally beneficial relationship with the host when they are in the intestine; however, when they escape from this environment, they can cause significant pathologies, including bacteremia and abscess formation, at multiple sites in the body [36, 38]. Additionally, species of this genus possess a large number of antibiotic resistance mechanisms and the highest resistance rates of all anaerobic pathogens. Clinically, *Bacteroides* species have shown increasing resistance to many antibiotics, including cefoxitin, clindamycin, metronidazole, carbapenems, and fluoroquinolones (e.g. gatifloxacin, levofloxacin and moxifloxacin) [38]. Similarly, an increase in the abundance of this biomarker has been related to obesity and low-grade inflammation [36, 39]. Future studies should strive to understand the pivotal role that this genus plays in its coexistence with *Blastocystis.*

Regarding other biomarkers, Audebert et al. [18] reported a positive association between *Blastocystis* and the genera *Faecalibacterium*, *Ruminococcus* and *Prevotella* and a negative association with *Bacteroides* abundance. Significantly, *Faecalibacterium* bacteria produce butyrate, which is considered one of the most important metabolites for maintaining human colon health because it represents the main energy source of colon epithelial cells. In addition, this metabolite has anti-inflammatory properties and participates in the regulation of gene expression, differentiation, and apoptosis in host cells [18, 40, 41]. Significant reductions in the abundance of these bacteria have been identified as markers of dysbiosis in patients with ulcerative colitis or Crohn’s disease [42–44].

Finally, evaluating the general composition of the microbial community between the two groups of participants (beta diversity), from the calculation of Bray-Curtis dissimilarity distances and the generation of a PCoA, despite the fact that no groupings related to the presence of *Blastocystis*; the existence of three groups is identified (Fig. 5). However, when analyzing the different variables of a biological and epidemiological nature that were initially collected, none was found to explain the grouping. It is probable that at the taxonomic level of phylum it is possible to show the conformation of certain groups, for which studies that involve a greater number of biological, sociodemographic and epidemiological variables are required to characterize these findings.

*Blastocystis* is considered by various authors and clinical practitioners to be a gastrointestinal pathogen, and it has been proposed as one of the mechanisms behind the pathophysiology involved in dysbiosis of intestinal microbiota [1]. Although longitudinal studies are required to determine the causal association between *Blastocystis* and changes at the level of the intestinal microbiota, our study suggests that this protozoan is part of a normal or healthy microbiota and is harmless and potentially beneficial to the carrier, contributing to an increase in microbiota richness, which is associated with human health [45]. Additionally, our results suggest that the presence of *Blastocystis* may be related to a decrease in the relative abundance of *Bacteroides* bacteria and an increase in *Faecalibacterium*, a relationship that is, potentially, involved in creating an anti-inflammatory environment in the human intestine that could decrease an individual’s susceptibility to diseases associated with inflammatory bowel processes. However, as we did not find statistically significant differences, we cannot conclude that *Blastocystis* dramatically increases or decreases beneficial bacteria. In this regard, we require a broader sampling to completely rule out this debate, at least in Colombian populations. Also, our population was mainly asymptomatic and frequently exposed to intestinal parasitism which might have biased our study and could explain the lack of statistical significance. Experimental studies aimed at evaluating the microbiome in its entirety (bacteriome, eukaryome and virome) and those using animal models, as well as prospective studies to evaluate the association and physiological mechanisms of changes in the abundance and diversity of these genera in the intestinal microbiota, are required. Additionally, the fact that, lately, some common luminal intestinal parasitic protists (CLIPPs), such as *Blastocystis*, have been found more often in healthy than diseased individuals supports the hypothesis that some parasites might be protective against disease [46]. For this reason, studies are needed to reveal the biological and physiological aspects associated with health and disease states and to uncover how these CLIPPs associate and interact with the other components of the intestinal microbiota.

## Conclusions

To our knowledge, this is the first study of a Colombian school-age population comparing the components of the intestinal microbiome using amplicon-based sequencing of *16S* rRNA genes and comparing individuals with and without *Blastocystis* colonization using molecular tests. The results suggest at least descriptively that there is a potential greater microbiome richness in those colonized by *Blastocystis*; therefore, the protozoan could be eventually considered beneficial or innocuous to intestinal health (at least in this studied population) [45, 47]. However, because statistical analysis did not find the results to be significant, further prospective studies are necessary to confirm this hypothesis. It is also evident that the presence of this protozoan is not related to dysbiosis at the intestinal level and that, on the contrary, its presence may be linked to a “normal” or “healthy” microbiota. The protozoan may fulfill a role within the ecology of the intestinal microbiota through its interaction with the other components. A potential relationship between the presence of this protozoan and a decrease in *Bacteroides* bacteria, as well as an increase in the relative abundance of *Faecalibacterium*, was identified including the high relative abundance of certain families, effects that may be associated with a healthy intestinal microbiota *via* the generation of an anti-inflammatory environment [42, 44, 48]. This study was descriptive and, therefore, no causal associations can be established. Future metagenomic studies, as well as longitudinal, prospective, and animal models’ studies, and also including the ST as variable, that facilitate the establishment of causality and association, are required to explain the relationships between this protozoan and changes in the intestinal microbiota and the physiological mechanisms involved. Similarly, a fundamental understanding of the relationships between this protozoan and the other components of the intestinal microbiota would require studies of the microbiome in its entirety, including the bacteriome, eukaryome and virome. Knowledge of the interactions among the microorganisms that make up this environment will help to reveal key functional information about the various physiological and pathogenic mechanisms involved in intestinal *Blastocystis* parasitism.

## Supplementary information

**Additional file 1: Table S1. **General statistics of amplicon-based sequencing data. Reads metrics from output sequence data (quality-approved, trimmed and filtered data) and mean number of preprocessed reads per index after the high-quality filtering approach.

**Additional file 2: Figure S1.** **a** Bar chart of relative abundance of phylum for *Blastocystis*-colonized, *Giardia*, co-infection by two species, and not colonized subjects. **b** Boxplots of observed OTUs richness and Shannon diversity indices for *Blastocystis*-colonized, *Giardia*, co-infection by two species, and non-colonized subjects. Statistical analyses were performed using the Kruskall-Wallis (KW) test to compare groups. Plotted are interquartile ranges (IQRs; boxes), medians (lines in the boxes), and the lowest and highest values within 1.5 times IQR from the first and third quartiles (whiskers above and below the boxes).

## Data Availability

The datasets generated and/or analyzed during the present study will be available in the European Nucleotide Archive (ENA) repository. Study accession number: PRJEB38738.

## Abbreviations

STs: Subtypes
qPCR: Quantitative polymerase chain reaction
QIIME: Quantitative insights into microbial ecology
ASV: Amplicon sequence variants
CLIPPs: Common luminal intestinal parasitic protists
