# Supplementary figures and images for: Microbiota characterization in Blastocystis-colonized and Blastocystis-free school-age children from Colombia
Source: Parasit Vectors. 2020 Oct 16;13:521. doi: 10.1186/s13071-020-04392-9 (PMC7565366; doi:10.1186/s13071-020-04392-9)

**a**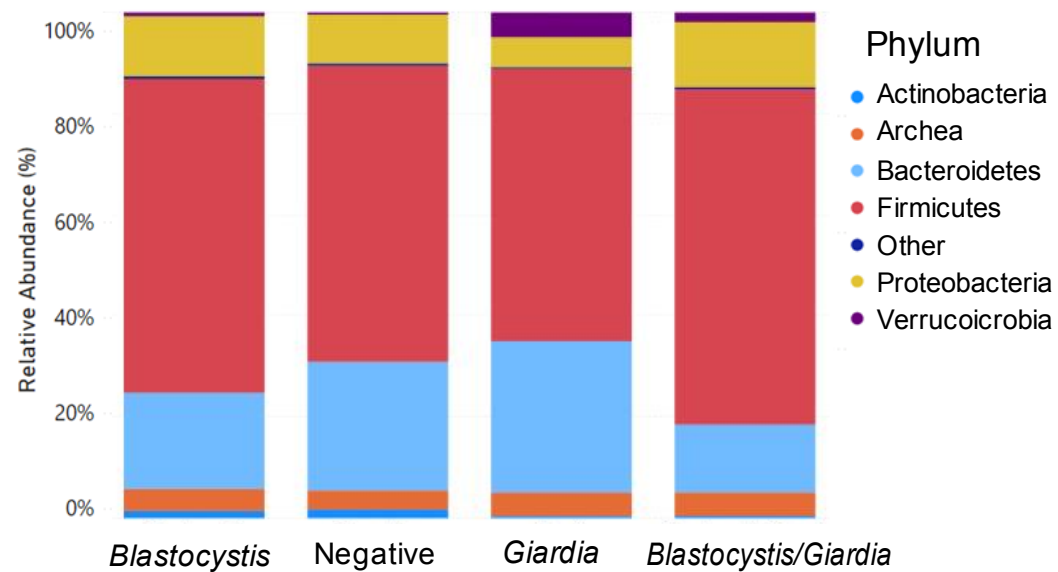**b**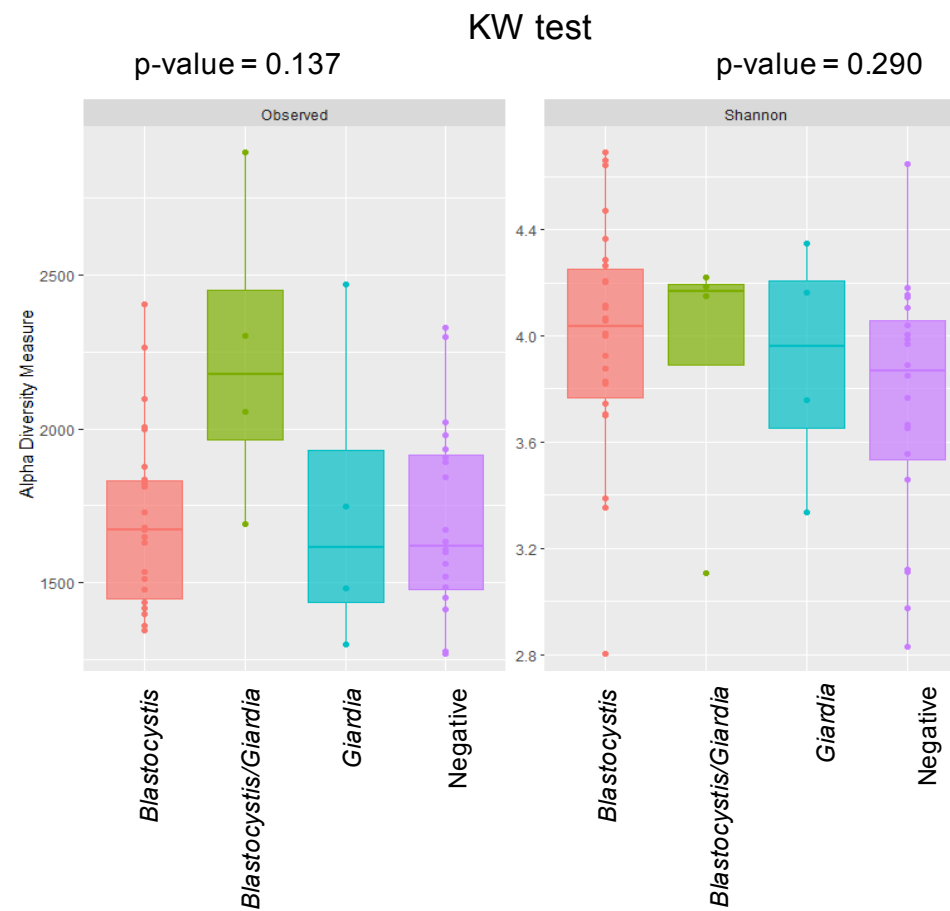

Supplement: Supplementary file 2 — Additional file 2: Figure S1. a Bar chart of relative abundance of phylum for Blastocystis-colonized, Giardia, co-infection by two species, and not colonized subjects. b Boxplots of observed OTUs richness and Shannon diversity indices for Blastocystis-colonized, Giardia, co-infection by two species, and non-colonized subjects. Statistical analyses were performed using the Kruskall-Wallis (KW) test to compare groups. Plotted are interquartile ranges (IQRs; boxes), medians (lines in the boxes), and the lowest and highest values within 1.5 times IQR from the first and third quartiles (whiskers above and below the boxes). [file 13071_2020_4392_MOESM2_ESM.pdf]
